# Relationship between physical activity and risk of depression in a married group

**DOI:** 10.1186/s12889-024-18339-7

**Published:** 2024-03-15

**Authors:** Rong Jing Ni, Ying Yu

**Affiliations:** Physical Education Institute, Liaoning Normal University, Dalian, 116029 China

**Keywords:** Physical activity, Depression risk, In-married group, Both sexes

## Abstract

**Background:**

Currently, there are many different findings on the relationship between physical activity and depression, and there may be differences between genders. This study therefore focused on gender differences to understand the relationship between physical activity behaviour and the risk of depression in married individuals.

**Methods:**

15607 married people in the China Family Panel Studies 2020 (CFPS 2020) were used to understand the relationship between physical activity and depression risk in different populations, and the chi-square test, Mann-Whitney U-test, and binary logistic regression were used to explore the relationship between physical activity and depression risk in the married population.

**Results:**

527 (6.64%) women were at high risk of depression and 365 (4.76%) men were at high risk of depression; physical activity was associated with the risk of depression in the married population, but after incorporating demographic and relevant cognitive variables, physical activity was negatively associated with the risk of depression in women (*OR* = 0.94, *P* < 0.01) but not statistically significant with the risk of depression in men (*OR* = 0.96, *P* > 0.05).

**Conclusion:**

Physical activity was directly related to the risk of depression in married women, but not in married men.

In recent years, mental health problems have been one of the major contributors to the global burden of disease. A WHO-commissioned study showed that depression and anxiety disorders increased by more than 25% globally during the pandemic [1]. Depression is a debilitating illness that affects individuals and families, and depressive states are not only associated with organic problems such as chronic low back pain and bouts of headache [2–4], but also with behavioral problems such as insomnia and idiosyncratic anger [5, 6], and are one of the factors contributing to the deterioration of family interactions [7]. Negative cognitive tendencies during depression make a person more likely to perceive lower marital satisfaction [8], which in turn triggers the breakdown of the marital relationship.

Many epidemiological studies convey the same message: marriage is good for health and divorce threatens physical and mental health [9, 10], but a closer look at individual differences reveals a more nuanced view. Turbulent marriages can breed depression and impair health, and marital intimacy and happiness are at risk when couples share unhealthy behaviors [11]. However, in most of our previous studies, only the relationship between marital status and depression has been discussed [12, 13], but the impact of marital quality and the marital relationship on people’s mental health has been neglected. For most people, post-divorce emotional distress is relatively short-lived [14], and very low marital quality within a marriage can, on the contrary, lead to lower life satisfaction for women [15]. Numerous studies have found that women who are troubled by poor marital relationships are at greater risk than men [9]. Women have a greater emotional response to negative marital behaviors than men [16] and are more likely to constantly evoke memories of stressful experiences, which in turn promote depression and inflammation [17]. Therefore, it is also necessary to pay attention to the depression status of different genders in the married population.

Some imaging evidence suggests that higher marital quality predicts lower levels of depression, especially among married women [18]. Even within specific populations, marital quality influences couples’ levels of depression as well as other psychological distress to some extent [19, 20]. In conclusion, it is necessary to include marital quality as an important factor influencing the mental health of couples in the study of depression in the married population. According to the 2022 survey data, China’s depression risk detection rate is as high as 10.6% [21]. In addition to this, the crude divorce rate in China has been increasing incrementally since 1979, reaching a peak of 3.4 per thousand in recent years in 2019 [22]. Therefore, in the face of the unpromising depression risk detection rate and divorce rate in China, it is necessary to explore the relationship between marital relationships and individual depression from a more nuanced perspective and to understand the role played by gender differences, to provide a theoretical basis for maintaining happy and harmonious intimate relationships and safeguarding the psychological health of family members.

In response to the issue of mental health promotion, many foreign studies have identified the positive effects of physical exercise on different groups [23, 24], and exercise prescription has begun to be applied in clinical studies of depression in different populations [25, 26]. A mixture of moderate-intensity aerobic and anaerobic exercise [27], positive yoga [28], tai chi [29], and numerous other forms of physical exercise are effective in the prevention and treatment of depression. Currently, numerous studies are exploring the factors influencing people’s physical activity behavior, of which marital status can often be of interest to researchers. Studies have shown that marital status has a negative effect on individuals’ participation in physical activity [30], with youth with spouses participating in physical activity significantly less frequently than youth without spouses [31]. However, it has also been suggested that good marital status can promote individuals’ participation in physical activity [32]. In some more in-depth studies, both marital support and stress have been found to be associated with more frequent exercise and the odds of walking, suggesting that the influence of marital quality in this is complex [33]. In addition to this, gender also differs in the sporting behavior of the married population. Married women tend to engage in less physical activity than married men [34]. And these subtle differences are not reflected in the unmarried group [35]. Although the role of marital quality is complex, what can be determined is that for the in-married group, marital quality is one of the important influences on their physical activity behavior.

In summary, the marital relationship is one of the most important factors influencing health behaviors and well-being [36], and it has an impact on depression levels as well as physical activity behaviors in the married group. Although an association between physical activity and depression has also been demonstrated in the unmarried group, the influence of spouses on each other’s emotional support as well as on health behaviors such as physical activity is specific to the in-marriage group. Therefore, marital quality plays a role in the relationship between depression and physical activity that distinguishes it from the unmarried group. While research on the relationship between physical activity and depression in China has focused on students and the elderly, there is limited research exploring the relationship between physical activity and depression in the married group from an intimacy perspective. Accordingly, this study uses the 2020 China Family Panel Studies (CFPS) as the sample source and focuses on the relationship between physical activity behavior and depressive mood in the married group based on cross-sectional data of the married group in China. The study also focuses on gender differences in the field of mental health and provides an empirical basis for reducing the risk of depression and improving psychological quality in the married population.

## Study design

### Data sources

This study uses data from the 2020 cross-section of the China Family Panel Studies (CFPS 2020) conducted by the Institute of Social Science Survey (ISSS) at Peking University, a large-scale, multidisciplinary social survey that focuses on family relationships, family dynamics, and health. The 2020 CFPS has a sample size of 28,590 individuals at the individual level. 28,590 individuals, with 15,607 complete samples retained for this study after sequentially removing 3,734 interrupted and incomplete interviews, and 9,249 samples that were inapplicable or missing for any of the questions related to this study.

### Selection of variables

All selection variables are shown in Table 1 below. Depressed mood was selected as the dependent variable measure, which was based on eight items from the Center for Epidemiological Studies Depression Scale (CES-D), entitled “Indicate how often various feelings or behaviors occurred in the past week”, including “I feel depressed”, “I find it hard to do anything”, “I don’t sleep well”, “I feel happy”, “I feel lonely”, “I feel like I’m not in a good mood”, “I feel lonely”, “I am happy with my life”, “I feel sad and upset” and “I don’t feel like I can go on with my life”. The scale has four levels, which are ‘Hardly ever (less than a day), some of the time (1–2 days), often (3–4 days), and most of the time (5–7 days)’, and are assigned values on a scale of 1 to 4(the reverse question was transposed), with higher total values indicating a greater risk of depression. The total scores for the 8 question items ranged from 8 to 32, with a median of 20 used as the cut-off value. When a number slightly larger than 20 was used as the cut-off value, there was no substantial change in the statistical results, indicating that the cut-off value of 20 was robust. Therefore, it was finally determined that those with a total score greater than 20 were in the high-risk group and the rest were in the low-risk group.

The question “frequency of physical exercise” was selected as the basis for the independent variable indicator, which is “excluding cycling and walking for the single purpose of going to and from work, how often did you participate in physical fitness and leisure activities in the past 12 months?“. According to the options, there are 8 groups, and “never participate” is assigned as 0, “on average less than once a month” is assigned as 1, “on average more than once a month, but less than once a week” is assigned as 2, “on average 1–2 times a week” is assigned as 3, “on average 3–4 times a week” is assigned as 4, “on average 5 times a week and more” is assigned as 5, “once a day” is assigned as 6, and “twice a day and more” is assigned as 7.

To understand the nature of the relationship between physical activity and the risk of depression in the in-marriage group, proximal factors that have a strong relationship with physical behavior and psychological status in the in-marriage group were also included in the study. According to existing studies, depression levels among Chinese residents are related to age, gender, marital status, work status, and relationship satisfaction with spouse [37, 38]. Urban-rural differences, educational level, economic status, and social status have also been shown to play an influence on physical activity and depression [39]. Depression as a psychological perception cannot be separated from the influence of subjective psychological evaluation indicators such as happiness, life satisfaction, and health perception [40, 41]. Therefore, by combining the above studies, it was decided that the four aspects of basic personal situation, intra-marital relationship, life feeling and social relationship would also be included in the study. The specific indicators were chosen as shown in Table 1 below. Among them, marital satisfaction, satisfaction with the other party’s economic contribution, satisfaction with the other party’s contribution to household chores, life satisfaction, self-perceived health, and subjective social status are level 5 indicators, with 1 representing the lowest degree and 5 representing the highest degree. Subjective well-being and subjective personhood are 11-level indicators, with 0 representing the lowest degree and 10 representing the highest degree.

**Table 1:** Demonstration of each variable

Dimensionality	Variables	Variable description	M ± SD
Basics	Risk of depression	1 = “Low risk”; 2 = “High risk”	1.68 ± 0.51
	Frequency of exercise	0 = “Never”; 1 = “On average, less than once a month”; 2 = “On average, more than once a month, but less than once a week”; 3 = “On average, 1–2 times a week”; 4 = “On average, 3–4 times a week”; 5 = “On average, 5 times a week and more”; 6 = “Once a day”; 7 = “Twice a day and more”	1.51 ± 2.36
	Age	1 = “Youth”; 2 = “Middle age”; 3 = “Old age”	1.88 ± 0.78
	Gender	1="Male”; 2="Female”	1.51 ± 0.50
	Place of residence	1="Town”; 2="Village”	1.49 ± 0.50
	Region	1="East”; 2="Central”; 3="West”; 4="North East”	2.22 ± 1.05
	Are highly educated	0="No”; 1="Yes”	0.12 ± 0.32
	Active status or not	0="No”; 1="Yes”	0.78 ± 0.42
	Children under 16 years of age	0="No”; 1="Yes”	1.41 ± 1.44
Marital relationship	Marital satisfaction	1 ∼ 5 assignment; 1 means very unsatisfied, 5 means very satisfied	4.48 ± 0.85
	Satisfaction with the economic contribution of the other party	1 ∼ 5 assignment; 1 means very unsatisfied, 5 means very satisfied	4.28 ± 0.10
	Satisfaction with each other’s contribution to household chores	1 ∼ 5 assignment; 1 means very dissatisfied, 5 means very satisfied	4.16 ± 1.13
Life feelings	Life satisfaction	1 to 5 assignment; 1 = “Very dissatisfied”, 5 = “Very satisfied”	4.14 ± 0.80
	Subjective well-being	0 ∼ 10 assignment; 0 is the lowest, 10 is the highest	7.57 ± 2.05
	Self-perceived health	1= “Unhealthy”; 2= “Fair”; 3= “Relatively healthy”; 4= “Very healthy”; 5= “very healthy”	3.00 ± 1.20
Social relations	Subjective human relations	0 ∼ 10 assignment; 0 is the lowest, 10 is the highest	7.13 ± 1.90
	Subjective social status	1 ∼ 5 assignment; 1 means very low, 5 means very high	3.16 ± 1.05

### Mathematical statistics

A chi-square test with the Mann-Whitney U test was used to compare groups using spss27.0 and a binary logistic regression analysis was conducted to explore the relationship between physical activity and the risk of depression in each gender. The test level was α = 0.05.

## Analysis of results

### Overview of the study population

As shown in Table 2, among the 15,607 study subjects, a total of 892 were at high risk of depression, including 527 women and 365 men, and the proportion of women at high risk of depression was higher than that of men. A chi-square test was carried out to stratify the populations and showed that the difference was not statistically significant for the female group only in terms of work status, and for the male group in terms of work status and children under 16 years of age, while the rest of the indicators were statistically significant.

The distribution of depression risk was similar between females and males in terms of age, place of residence, region, and education, with higher levels of depression risk in the western region, rural areas, low education levels, and middle-aged and older groups. Only the female group had a statistically significant risk of depression in terms of children under 16 years of age, and the married female group with no children under 16 years of age showed higher levels of depression. In terms of work status, although neither was statistically significant, the depression levels were higher in the unemployed male group compared to the female group.

**Table 2:** Comparison of populations at risk of depression

Variables	Group	Female			Male		
		Number of people	Number of people at high risk	χ2	Number of people	Number of people at high risk	χ2
Age	Youth	3216	130 (4.04%)	63.20***	2579	86 (3.33%)	17.78***
	Middle age	2964	232 (7.83%)		2907	164 (5.64%)	
	Elderly	1757	165 (9.39%)		2184	115 (5.27%)	
Place of residence	Towns	4097	217 (5.30%)	24.65***	3879	130 (3.35%)	34.30***
	Countryside	3840	310 (8.07%)		3791	235 (6.20%)	
Region	East	2589	119 (4.60%)	28.83***	2557	79 (3.09%)	33.41***
	Central	1994	139 (6.97%)		1911	89 (4.66%)	
	Western	2247	186 (8.28%)		2227	148 (6.65%)	
	North East	1107	83 (7.50%)		975	49 (5.03%)	
Higher Education	No	7002	497 (7.10%)	20.13***	6751	342 (5.07%)	11.73***
	Yes	935	30 (3.21%)		919	23 (2.50%)	
Are you in business	No	2357	169 (7.17%)	1.52	1150	61 (5.30%)	0.89
	Yes	5580	358 (6.42%)		6520	304 (4.67%)	
Have children under 16 years of age	No	2424	199 (8.21%)	13.87***	2421	126 (5.20%)	1.55
	Yes	5513	328 (5.95%)		5249	239 (4.55%)	
Total		7937	527 (6.64%)		7670	365 (4.76%)	

Note: ***P < 0.001

### Depression risk based on comparison of indicators

Mann-Whitney U test was conducted to understand the relationship between the risk of depression and various psycho-cognitive factors and the comparative results are shown in Table 3, the difference in subjective social status in the married group of females was not statistically significant and there was a high significant difference in the rest of the variables. The male in-marriage group had highly significant differences in all variables.

**Table 3:** Depression risk based on comparison of indicators

Variables	Female			Male		
	Low risk	High risk	Z	Low risk	High risk	Z
Frequency of exercise	3997.39	3569.81	-5.02***	3855.32	3438.92	-4.14***
Marital satisfaction	4031.79	3086.17	-10.28***	3867.54	3194.22	-7.52***
Satisfaction with the economic contribution of the other party	4033.72	3059.06	-10.23***	3872.55	3093.94	-7.76***
Satisfaction with each other’s contribution to household chores	4009.04	3405.97	-6.13***	3871.39	3117.11	-7.81***
Life satisfaction	4056.34	2740.90	-13.08***	3891.71	2710.43	-10.26***
Subjective well-being	4069.16	2560.73	-14.90***	3914.80	2248.34	-14.33***
Self-perceived health	4078.07	2435.36	-16.74***	3900.04	2543.79	-12.05***
Subjective Human Relations	4005.28	3458.92	-5.40***	3861.82	3308.75	-4.76***
Subjective social status	3967.57	3989.17	-0.22	3855.34	3438.38	-3.75***
Total number of people	7410	527		7305	365	

Note: ***P < 0.001

### Gender-based comparison of the indicators

To explore whether there are gender differences in each indicator, gender was used as the dependent variable (1 = male, 2 = female), and separate comparisons were made between the low-risk group of depression and the high-risk group of depression again separately, and the results of these comparisons are shown in Table 4 below. In the low-risk group, gender differences were significant to varying degrees in frequency of exercise, marital satisfaction, satisfaction with the other person’s financial contribution, satisfaction with the other person’s household contribution, subjective well-being, and self-perceived health. In the high-risk group, gender differences were significant to varying degrees in marital satisfaction, satisfaction with each other’s financial contribution, satisfaction with each other’s housework contribution, self-perceived health, and subjective social status. It is noteworthy that the frequency of exercise showed highly significant gender differences in the low-risk group, while no significant gender differences were seen in the high-risk group. Once again, this suggests that it is necessary to differentiate between genders for the study of the relationship between physical activity and the risk of depression.

**Table 4:** Comparison of gender-based indicators

Variables	Low risk			High risk		
	Female	male	Z	Female	male	Z
Frequency of exercise	7272.50	7444.73	-2.92**	442.03	452.95	-0.86
Marital satisfaction	6688.42	8037.21	-23.23***	405.89	505.13	-6.11***
Satisfaction with the economic contribution of the other party	6800.99	7923.02	-18.10***	413.71	493.84	-4.79***
Satisfaction with each other’s contribution to household chores	6172.96	8560.07	-37.77***	395.70	519.85	-7.41***
Life satisfaction	7301.52	7415.29	-1.68	439.56	456.52	-0.98
Subjective well-being	7285.68	7431.36	-2.13*	445.74	447.59	-0.11
Self-perceived health	7096.20	7623.57	-7.96***	425.96	476.16	-3.06**
Subjective Human Relations	7350.77	7365.33	-0.21	446.90	445.92	-0.06
Subjective social status	7345.35	7370.84	-0.39	464.21	420.92	-2.54*
Total number of people	7410	7305		527	365	

### Relationship between physical activity and risk of depression in married groups

To further understand the relationship between the frequency of physical activity and risk of depression, binary logistic regression analyses were conducted with risk of depression as the dependent variable (1 = low risk, 2 = high risk) and frequency of exercise as the independent variable, as well as indicators screened by previous between-group comparisons, and the final results are shown in Table 5. The association of frequency of exercise with depression in women was statistically significant (*OR* = 0.94, 95%*CI* = 0.89 ∼ 0.98, *P* < 0.01), but the association with depression in men was not statistically significant (*OR* = 0.96,95%*CI* = 0.92 ∼ 1.02, *P* = 0.176). Compared with young women, middle-aged women, and older women were at higher risk of depression, and the association between age and depression was not significant in the male population. For both women and men, the risk of depression was higher in the rural group compared to the urban group, and the risk of depression was higher in the rest of the region compared to the eastern region.

In terms of related cognitive indicators, life satisfaction, subjective well-being, and self-perceived health were significantly associated with the risk of depression in both groups, with higher levels of life satisfaction, subjective well-being, or self-perceived health being associated with a lower risk of depression. In addition to this, marital satisfaction was associated with the risk of depression in women, and the higher the marital satisfaction, the lower the risk of depression, while the risk of depression in men was not significantly associated with marital satisfaction. The risk of depression in the male group was associated with subjective human relations, whereas the risk of depression in the female group was not associated with subjective human relations.

**Table 5:** Binary logistic regression analysis of exercise and risk of depression [*OR* (*OR* 95% *CI*)

Variables	Female	Male
Frequency of exercise	0.94(0.89 ∼ 0.98)**	0.96(0.92 ∼ 1.02)
Middle age	1.38(1.07 ∼ 1.78)*	1.25 (0.93 ∼ 1.66)
Elderly	1.87(1.40 ∼ 2.49)***	1.20 (0.87 ∼ 1.67)
Countryside	1.30(1.06 ∼ 1.59)*	1.59 (1.25 ∼ 2.03)***
Central	1.55(1.19 ∼ 2.03)**	1.55 (1.13 ∼ 2.14)**
Western	1.69(1.31 ∼ 2.19)***	1.73 (1.28 ∼ 2.33)***
North East	1.67(1.23 ∼ 2.28)**	2.00 (1.36 ∼ 2.94)***
Have any children under 16 years of age		
Yes	0.85(0.69 ∼ 1.05)	
Educated or not		
Yes	0.78(0.51 ∼ 1.18)	0.84 (0.52 ∼ 1.33)
Marital satisfaction	0.85(0.76 ∼ 0.95)**	0.90 (0.78 ∼ 1.05)
Economic satisfaction of the other party	0.92(0.83 ∼ 1.02)	0.95 (0.83 ∼ 1.08)
Satisfaction with each other's household chores	1.04(0.95 ∼ 1.14)	0.88 (0.76 ∼ 1.02)
Life satisfaction	0.70(0.62 ∼ 0.80)***	0.73 (0.63 ∼ 0.85)***
Subjective well-being	0.82(0.78 ∼ 0.87)***	0.75 (0.71 ∼ 0.80)***
Self-perceived health	0.61(0.56 ∼ 0.67)***	0.64 (0.58 ∼ 0.71)***
Subjective human relations	1.04(0.98 ∼ 1.09)	1.08 (1.02 ∼ 1.15)*
Subjective social status		1.08 (0.96 ∼ 1.22)

Note: *P < 0.05, **P < 0.01, ***P < 0.001

## Discussion

The study found that the frequency of physical activity was significantly correlated with the risk of depression in the married group. After incorporating demographic indicators and some subjective cognitive indicators, the risk of depression among married women remained associated with exercise frequency, but the relationship between the risk of depression and exercise frequency among married men was not statistically significant.

### Gender differences in depression risk

In this study, the gender differences regarding the risk of depression in the married group were the same as in other national studies, with women having a higher rate of depressive disorders than men [42–44], and the reasons for this can be explored in three main ways based on previous studies.

First, physiological reasons. For example, women have lower levels of 5-hydroxytryptamine, which is a neurotransmitter that gives pleasure [45], or reproductive depression caused by hormonal changes such as the menstrual cycle, pregnancy, and menopause.

Secondly, socio-sexual factors. Women under social gender roles have higher stress levels and feelings of helplessness [46] and are more likely to fall into a contemplative pattern of repressed emotions. It has been found that women need to constantly internalize anger and frustration under the pressure of traditional gender perceptions, which leads to their higher rates of depressive disorders or negative emotions [47]. This view can be reflected in many ways. For example, society’s excessive attention to women’s looks and body image [48]; the fact that women are less likely than men to be selected for promotion and prestigious positions throughout their careers [49]; and society’s expectation that women should be able to play the roles of good wives and mothers [50] are all events that intensify women’s stress in life and affect their mental health. In the process of modern gender concepts emphasizing equality between men and women, gender equalization will protect girls’ mental health by alleviating the conflict of gender concepts and female inferiority in the transition period [51]. Therefore, the regulation of gender concepts has a non-negligible role in women’s mental health. However, some empirical studies have shown that gender role stereotypes are reinforced when people enter marriage [52, 53] and continue into parenthood [54]. In addition to this, according to the gender display perspective, social gender role norms anticipate male and female behavior, and when a woman deviates from or violates this expectation, to balance the couple’s relationship, she maintains a more traditional view of gender and takes on more domestic work as compensation [55, 56]. Therefore, married women are more likely to favor traditional gender concepts.

In addition to this, although the effect of two marital indicators, financial satisfaction with the other person and satisfaction with the other person’s household chores, on the risk of depression is not statistically significant, it has been shown that perceived fairness in the division of household labor is negatively correlated with depression among working married women [57] and has been shown to alleviate women’s postpartum depressive symptoms [58]. Economic satisfaction has also been shown to moderate levels of perceived family support depression in some populations [59]. Some other studies have also suggested that when men earn more than their spouses, provide for the family well, and the dual role of the wife is difficult to balance, men would prefer women to take on traditional roles and fulfill traditional family responsibilities such as housework and childcare [60]. In conclusion, the two indicators of financial satisfaction with the other person and satisfaction with the other person’s housework may also have an impact on depression among married women in some cases.

Third, men’s depressive symptoms are more likely to be overlooked when self-reported or diagnosed [61]. Gender roles not only influence women’s mental health but also men’s self-reports of depressive symptoms. Although men are diagnosed with depression at half the rate of women in existing studies, they die by suicide three to four times more often than women, and the process of gender role socialization affects the way some boys and men express depression [62]. There are several reasons for this, such as the tension between traditional masculine norms and the expression of emotions, and the tendency to reach out for help only when internal resources are exhausted and depressive symptoms are severe [63]. In addition to this, the diagnostic labels for depressive symptoms include words such as low mood, irritability, and restlessness that are not recognized because they go against the norms of their ideal masculinity, preferring instead to acknowledge stressfulness, overwhelm, etc. [64].. In sum, traditional norms of masculinity prevent men from actively reporting depressive symptoms, resulting in lower rates of detection of depressive symptoms in men than in women in numerous self-report studies.

### Relationship between physical activity and depression

The extremely significant correlation between frequency of physical activity and risk of depression in both the married male and married female samples, before the inclusion of other variables, is consistent with the findings of several previous studies. There is now a lot of evidence on the relationship between physical activity and depression, mainly in two areas.

On the one hand, it is believed that physical activity has a depressive inhibitory effect. For example, in animal experiments, physical exercise can promote the elevation of the levels of antioxidant enzymes, brain-derived neurofactors, etc., and reduce the level of cortisol with the reduction of inflammation in brain tissue [65]. Neurobiology has shown that exercise increases the volume of grey matter in the brain, improves the microstructure of white matter, and is associated with functional connectivity in brain regions associated with major depression [66]. In addition to this, the social benefits of physical activity can also intervene in depression levels. For example, physical activity exerts depressive inhibition by strengthening an individual’s self-concept and enhancing perceived levels of social support [67]. Sedentary behavior is now recognized as a causative factor for negative emotions or psychological disorders in numerous studies [68–70], and in studies of Chinese residents, regular, sustained physical activity [71] or moderate to high-intensity physical activity [72] can serve as a protective factor against depressive symptoms. Physical activity is one of the most important ways to effectively reduce sedentary time and increase the level of physical activity, so physical activity behaviors have been considered as one of the factors to reduce the level of depression and promote mental health.

On the other hand, it has also been suggested that negative emotions can reduce people’s motivation to be physically active. Physical activity or fitness may prevent depression, apparently because participants with higher levels of physical activity and fitness were less likely to report high levels of depressive symptoms [73]. That is, depressive symptoms may inhibit people’s motivation to engage in physical activity. This idea is also reflected in older age groups, where greater social isolation in older men and women is associated with reduced daily physical activity and increased sedentary time, and thus differences in physical activity may lead to increased health risks [74].

### Gender differences in physical activity and depression correlations

After incorporating other relevant indicators, the correlation between physical activity and the risk of depression in married women remained significant, but the association with the risk of depression in men was not statistically significant, which was analyzed in the context of the aforementioned differences between the sexes for the following reasons.

First, the biological mechanisms generated by physical activity are more prominent for the female population. In an experiment on rats with vascular dementia, it was found that treadmill exercise, whether voluntary or mandatory, up-regulated their dopamine and 5-hydroxytryptamine levels to protect cognition [75], which has a compensatory mechanism for the lower turnover of 5-hydroxytryptamine levels in women [76]. In addition to this, under the same workload, the sympathetic nerve activity is more active in females, and the levels of norepinephrine, epinephrine, and glucose are significantly higher than those of males, whereas there is no difference between the two indicators at rest [77]. Thus, the moderating effect of physical activity on certain physiological levels was more prominent in the female group than in the male group, and the correlation between physical activity and the risk of depression in women became more significant.

Secondly, men under marital structure face societal pressures that distinguish them from women. The traditional marriage script requires men to take on the role of “breadwinner,” which gives them decision-making power in the family but also puts them under corresponding family pressure. For example, when unemployment affects people’s psychological well-being by depriving them of social contacts outside the home and the potential benefits of personal status [78], gender roles have a mediating effect on the anxiety of unemployed females, but can exacerbate negative emotions in groups of unemployed males [79]. In marital relationships, the female sample reported higher self-related relationship stress, while the male sample reported higher external stress, including transport problems, work stress, relationships with leaders, and the environment [80]. Therefore, objective pressures from external sources hardly make physical activity directly related to their mood states but rather manifest themselves in moderating depression levels by enhancing personal perceived factors such as well-being, life satisfaction, and improving interpersonal relationships.

Again, differences in motivation and expectations for physical activity between the sexes resulted in different interventions for depression. In a survey of gender differences, women’s motivations for physical activity mostly focused on direct physical benefits such as appearance, improved health, and weight control, whereas men wanted to socialize, gain social recognition, enjoyment, and invisible benefits such as strength through physical activity [81]. Women’s expectations of physical activity are easier to obtain and quantify than men’s, and a good exercise experience has a more direct effect on negative emotions, whereas men’s expectations of exercise require a longer perceptual process and are ambiguously measured, making it difficult to obtain a definitive emotional experience, which resulted in a nonsignificant direct correlation between physical activity and depression in men.

Finally, lower self-reported rates of high depression risk among married men resulted in a less significant effect of physical activity. The marital relationship is one of the most important factors influencing the psychological state of both spouses. In most national samples, married groups are more likely to be happy than unmarried groups, and in-marriage status is a favorable indicator of well-being indices in men, but not in female samples [82]. Upon entering marital status, both partners receive care and support from their spouses, and women provide much more care and support than they receive [83]. Data from a survey in rural China showed that unmarried men had higher levels of depression, aggression, low self-esteem, and suicidal tendencies that worsened with age, compared to married men who remained stable [84]. However, tendencies such as men’s reluctance to disclose emotional problems to outsiders versus resolving them on their own, influenced by gender roles [85], can also affect the veracity of their self-reports. This somewhat narrows the sample of men at high risk of depression, which in turn is associated with less than significant correlations with some of the indicators.

## Conclusion

Although studies have confirmed the relationship between physical activity and depression levels in various populations, as well as studies that have taken marital status into account, there is a limited empirical basis for studying the married group in terms of gender differences. The present study demonstrates a negative relationship between physical activity and depression in marital relationships, especially with depression levels in married women, and enriches the research on positive psychology and women’s psychology. This is of theoretical significance to help in-married women alleviate their negative emotions and enhance the happiness of their marital life. Although the relationship between physical activity and the level of depression in married men was not significant when other variables were added, the depressive state of the spouse may affect the fulfillment of family functioning and reduce marital satisfaction, so physical activity may have a role in play in dissipating some of the emotional problems and maintaining family harmony.

Based on this, it is appealed to all sectors of society to pay more attention to women’s mental health and the positive benefits of physical exercise for married women, promote the improvement of the social support system for women’s participation in sports, and enrich material, opportunity, and cultural resources. Actively promoting married women with dual family and social responsibilities with development pathways to release negative emotions and enhance their sense of well-being and fulfillment through physical exercise. Advocating for attention to the impact of marriage quality on the psychological state of both genders, we try to increase emotional links and mobilize the family atmosphere through regular physical exercise, especially sports activities that require the joint participation of both parties and actively play the role of intimate relationships in regulating mental health.

## Data Availability

Firstly, the CFPS data are already in the public domain and can be accessed directly at the following website: www.isss.pku.edu.cn/cfps/sjzx/gksj/index.htm. Secondly, the programs written by the users themselves during data processing and data analysis are the users’ achievements, and CFPS does not have any restrictions on this.

## References

[1] World Health Organization. Opening address by the Director-General of WHO to the World Economic Forum panel on mental health in the workplace on 18 January 2023. https://www.who.int/zh/director-general/speeches/detail/who-director-general-s-opening-remarks-at-the-mental-health-at-work-panel--world-economic-forum---18-january-2023

[2] Astrid F, Marion H, Lutz G (2014). Symptoms of depression impact the course of lung function in adolescents and adults with cystic fibrosis. BMC Pulm Med.

[3] Santos OD, Lilianel VFM, Rute SMS (2019). The impact of anxiety and depression on the outcomes of chronic low back pain multidisciplinary pain management-a multicenter prospective cohort study in pain clinics with one-year follow-up. Pain Med.

[4] Zwbenholzer K, Lechner A, Broessner G (2016). Impact of depression and anxiety on burden and management of episodic and chronic headaches - a cross-sectional multicentre study in eight Austrian headache centers. J Headache Pain.

[5] Rumble ME, McCall WV, Dickson DA (2020). An exploratory analysis of the association of circadian rhythm dysregulation and insomnia with suicidal ideation throughout treatment in individuals with depression, insomnia, and suicidal ideation. J Clin Sleep Med.

[6] Crisan SM, Nechita DM (2021). Maladaptive emotion regulation strategies and trait anger as predictors of depression severity. Clin Psychol Psychother.

[7] Wong JJ, Frost ND, Timko C (2020). Depression and family arguments: disentangling reciprocal effects for women and men. Fam Pract.

[8] Joelle LM, Gotlib IH (2019). Depression: a cognitive perspective. Clin Psychol Rev.

[9] Kiecolt JK (2018). Marriage, divorce, and the immune system. Am Psychol.

[10] Sbarra DA, Law RW, Portley RM (2011). Divorce and death: a meta-analysis and research agenda for clinical, social, and health psychology. Perspect Psychol Sci.

[11] Bourassa KJ, Sbarra DA, Whisman MA (2015). Women in very low-quality marriages gain life satisfaction following divorce. J Fam Psychol.

[12] Chen H, Li HW, Li XH (2023). Prevalence and factors affecting anxiety and depression among patients at Sanya Square Cabin Hospital during the COVID-19 epidemic in Hainan Province. Chin J Dis Control Prev.

[13] Lu WY, Zhang SX, Zhu JF (2023). A study on the correlation between health literacy and depression among occupational groups in selected enterprises in Shanghai. J Env Occup Med.

[14] Sbarra DA, Emery RE, Beam CR, Ocker BL (2013). Marital dissolution and major depression in midlife: a propensity score analysis. Clin Psychol Sci.

[15] Sbarra DA, Hasselmo K, Bourassa KJ (2015). Divorce and health: beyond individual differences. Curr Dir Psychol Sci.

[16] Kiecolt JK, Newton TL (2001). Marriage and health: his and hers. Psychol Bull.

[17] Ottaviani C, Thayer JF, Verkuil B (2016). Physiological concomitants of perseverative cognition: a systematic review and meta-analysis. Psychol Bull.

[18] Ma S, Zhang J, Wang L (2021). Efficient brain connectivity reconfiguration predicts higher marital quality and lower depression. Soc Cogn Affect Neurosci.

[19] An H, Chen C, Du R (2021). Self-efficacy, psychological distress, and marital quality in young and middle-aged couples facing lymphoma: the mediating effect of dyadic coping. Psychooncology.

[20] Maria CRG, Cody SH, Julia HTE (2023). Sexual dysfunction, depression, and marital dissatisfaction among Brazilian couples. J Sex Med.

[21] Science web. 2022 edition of the Blue Book of Mental Health released. 2023. https://news.sciencenet.cn/sbhtmlnews/2023/2/373371.shtm?id=373371. Accessed 24 Nov 2023.

[22] Sina. web. 3,928,000 pairs! 2023 Semi-Annual National Marriage Data. 2023. https://k.sina.com.cn/article_5880306529_15e7e5b61001015bzm.html. Accessed 24 Nov 2023.

[23] Dangelantoni M, Collins JL, Manchia M (2022). Physical exercise, depression, and anxiety in 2190 affective disorder subjects. J Affect Disord.

[24] Ricardo ALDS, Isabella RD, Lucas RSDO (2021). Molecular mechanisms of physical exercise on depression in the elderly: a systematic review. Mol Biol Rep.

[25] Ross RE, Vanderker CJ, Saladin ME (2023). The role of exercise in the treatment of depression: biological underpinnings and clinical outcomes. Mol Psychiatry.

[26] James AB, Alan R (2023). Exercise as a therapeutic modality for the prevention and treatment of depression. Prog Cardiovasc Dis.

[27] Felipe BS, Davy V, Simon R (2017). Exercise for depression in older adults: a meta-analysis of randomized controlled trials adjusting for publication bias. Braz J Psychiatry.

[28] Liu WM, Liu J, Ma L (2022). Effect of mindfulness yoga on anxiety and depression in early breast cancer patients received adjuvant chemotherapy: a randomized clinical trial. J Cancer Res Clin Oncol.

[29] Xie T, Song J, Zhu JF (2021). The effectiveness of Tai Chi on the depressive symptom of young adults with subthreshold depression: a study protocol for a randomized controlled trial. Trials.

[30] Zhang R (2014). Economic model and empirical test of sports demand and consumption. Sports Sci.

[31] Liu J, Lai S, Zhang Y. Social stratification of youth physical activity. Youth Res. 2023; (05): 56–68.

[32] Downward P (2007). Exploring the economic choice to participate in sport: results from the 2002 general household survey. Int Rev Appl Econ.

[33] Thomas PA, Richards EA, Forster AK (2022). Is marital quality related to physical activity across the life course for men and women. J Aging Health.

[34] Nomaguchi KM, Bianchi SM (2004). Exercise time: gender differences in the effects of marriage, parenthood, and employment. J Marriage Fam.

[35] Rapp I, Schneider B (2013). The impacts of marriage, cohabitation and dating relationships on weekly self-reported physical activity in Germany: a 19-year longitudinal study. Soc Sci Med.

[36] Umberson D, Crosnoe R, Reczek C (2010). Social relationships and health behavior across the life course. Annu Rev Sociol.

[37] Yang L, Zong ZH, Yi YY (2023). Study on the depression status and influencing factors of middle-aged and elderly women in rural China. Chin Gen Pract.

[38] Dong P, Ni ZJ, Zhao KQ et al. A survey of depression in the population during the novel coronavirus pneumonia epidemic. Chin Ment Health J. 2020,34(08):710–4.

[39] Fang L, Guo J (2019). Does physical activity promote health equity? - -Effects of physical activity on depression risk among urban and rural residents in China. China Sport Sci.

[40] Li Z, Zhang JY, Zheng FR (2023). Relationship between social integration status and risk of depressive symptoms among non-beijing express and takeaway workers in Beijing. Med Soc.

[41] Liu Y, Song XM (2023). Impact of disability status on depressive symptoms among the elderly in China. Med Soc.

[42] Özdin S, Bayrak ÖŞ (2020). Levels and predictors of anxiety, depression and health anxiety during COVID-19 pandemic in Turkish society: the importance of gender. Int J Soc Psychiatry.

[43] Hyde JS, Mezulis AH (2020). Gender differences in depression: biological, affective, cognitive, and sociocultural factors. Harv Rev Psychiatry.

[44] Joan SG, Yang K, Ferri CV (2017). The gender difference in Depression: are Elderly Women at Greater Risk for Depression Than Elderly. Men? Geriatr.

[45] Christine YN, Mark GM, Benjamin SA (2017). The role of 5-HT receptors in depression. Mol Brain.

[46] Mayor E (2015). Gender roles and traits in stress and health. Front Psychol.

[47] Kelly O. Shame, depression, and social melancholy. Sophia. 2020;59(1):31–38. 10.1007/s11841-020-00771-y

[48] Len JT, Luis RG, David BB (2018). Meritocracies or masculinities? The differential allocation of named professorships by gender in the academy. J Manage.

[49] Ramseyer WV, Gillen MM, Cahill L (2019). Body appreciation, anxiety, and depression among a racially diverse sample of women. J Health Psychol.

[50] Peersen S. The good, the bad and the ‘good enough mother’on the UK parenting forum Mumsnet. Womens Stud Int Forum. 2016:5932–8. 10.1016/j.wsif.2016.09.004

[51] Cheng C, Wang K (2020). Regional gender equalisation and adolescent mental health. J Xi’an Jiaotong Univ (Soc Sci Edit.

[52] Brooks C, Bolzendahl C (2004). The transformation of US gender role attitudes: cohort replacement, social-structural change, and ideological learning. Soc Sci J.

[53] Van EM, Baxter J, Buchler S (2010). A stalled revolution? Gender role attitudes in Australia, 1986–2005. J Popul Res.

[54] Endendijk JJ, Derks B, Mesman J (2018). Does parenthood change implicit gender-role stereotypes and behaviors?. J Marriage Fam.

[55] Brines J (1994). Economic dependency, gender, and the division of labor at home. Am J Sociol.

[56] Greenstein TN (2000). Economic dependence, gender, and the division of labor in the home: a replication and extension. J Marriage Fam.

[57] Claffey ST, Mickelson KD (2009). Division of household labor and distress: the role of perceived fairness for employed mothers. Sex Roles.

[58] Hwang W, Jung E, Shaw AV (2020). Paid leave and maternal depressive symptoms after childbirth: the moderating role of perceived fairness of the division of household labor. Fam Soc.

[59] Li Y, Zhao N, Zhao P (2022). The role of depression and economic satisfaction between family support and hope in older hypertensive patients. Chi J Prev Med.

[60] Adler MA, April B. East-West differences in attitudes about employment and family in Germany. Sociol Q. 1996:37245–60. 10.1111/J.1533-8525.1996.TB01748.X

[61] Rachel SH, Janet HS, Lyb AY (2017). Gender differences in depression in representative national samples: Meta-analyses of diagnoses and symptoms. Psychol Bull.

[62] Swetlitz N (2021). Depression’s Problem with men. AMA J Ethics.

[63] Oliffe JL, Phillips MJ (2008). Men, depression and masculinities: a review and recommendations. J Mens Health.

[64] Seidler ZE, Dawes AJ, Rice SM (2016). The role of masculinity in men’s help-seeking for depression: a systematic review. Clin Psychol Rev.

[65] Lucas RSDO, Frederico SMM, Isabella RD (2022). An overview of the molecular and physiological antidepressant mechanisms of physical exercise in animal models of depression. Mol Biol Rep.

[66] Repple J, Opeln N (2021). Sport and physical exercise in unipolar depression: Prevention, therapy, and neurobiological mechanisms of action. Nervenarzt.

[67] Zhang JL, Zheng S, Hu ZZ (2022). The Effect of Physical Exercise on Depression in College students: the Chain Mediating Role of Self-Concept and Social Support. Front Psychol.

[68] Huang Y, Li L, Gan Y (2020). Sedentary behaviors and risk of depression: a meta-analysis of prospective studies. Transl Psychiatry.

[69] Lee E, Kim Y (2019). Effect of university students’ sedentary behavior on stress, anxiety, and depression. Perspect Psychiatr Care.

[70] Wang X, Li Y, Fan H (2019). The associations between screen time-based sedentary behavior and depression: a systematic review and meta-analysis. BMC Public Health.

[71] Tian F, Yang X, Xu F (2023). Physical activity and its fluctuations in relation to depressive symptoms: a national longitudinal study among Chinese adults. J Affect Disord.

[72] Wu KG, Chen SJ, Hu YN (2023). The relationship between physical activity and depression among community-dwelling adults in Wuhan, China. Front Psychiatry.

[73] Johnson W, Mortensen EL, Kyvik KO (2020). Gene-Environment Interplay between Physical Exercise and Fitness and Depression Symptomatology. Behav Genet.

[74] Schrempft S, Jackowska M, Hamer M, Steptoe A (2019). Associations between social isolation, loneliness, and objective physical activity in older men and women. BMC Public Health.

[75] Zhang LL, Fan YZ, Kong XY (2020). Neuroprotective effect of different physical exercises on cognition and behavior function by dopamine and 5-HT level in rats of vascular dementia. Behav Brain Res.

[76] Paul W, Lesley AH, Webster M et al. Gender differences and effects of age on 5-hydroxytryptamine (5-HT) turnover: implications for irritable bowel syndrome (IBS). Gastroenterology. 2001;120.

[77] Lehmann M, Berg A, Keul J (2004). Sex-related differences in free plasma catecholamines in individuals of similar performance ability during graded ergometric exercise. Eur J Appl Physiol Occup Physiol.

[78] Frances MR, Zhao LS, Connie RW (2005). Psychological and physical well-being during unemployment: a meta-analytic study. J Appl Psychol.

[79] Paula A, Ana MU, Pilar M (2020). Social inequalities in health: duration of unemployment unevenly effects on the health of men and women. Eur J Public Health.

[80] Estlein R, Lavee Y (2021). Effect of daily stress on desire for physical proximity and emotional closeness. J Fam Issues.

[81] Ivanovic M, Ivanovic U (2018). Gender differences during adolescence in the motives for physical exercise, depression, anxiety and stress. Ex Qual Life.

[82] Hori M (2018). Gender differences in happiness: the effects of marriage, social roles, and social support in East Asia. Appl Res Qual Life.

[83] Claire AE, Judith SB. Psychology: about women. Shanghai People’s Publishing House; 2021. p. 202.

[84] Zhou X, Hesketh T (2017). High sex ratios in rural China: declining well-being with age in never-married men. Philos Trans R Soc Lond B Biol Sci.

[85] Rice SM, Oliffe JL, Kealy D (2020). Men’s help-seeking for depression: attitudinal and structural barriers in symptomatic men. J Prim Care Community Health.

